# Supplementary material for: A set of multi-entry identification keys to African frugivorous flies (Diptera, Tephritidae)
Source: Zookeys. 2014 Jul 24;(428):97–108. doi: 10.3897/zookeys.428.7366 (PMC4143993; doi:10.3897/zookeys.428.7366)
Supplement: Supplementary material 6 — Key to Ceratitis [file zookeys-428-097-s006.zip › SF6_ZooKeys_key to Ceratitis/key/SF6_ZooKeys_key to Ceratitis/Media/Html/Ceratitis paradumeti .htm]

Ceratitis (Ceratalaspis) paradumeti sp


***Ceratitis*** **(*Ceratalaspis*)
*paradumeti* De Meyer**

*Ceratitis* (*Ceratalaspis*)
*paradumeti* De Meyer, 1998 : 283.

Body
length: 6.00 (5.90-6.10) mm; wing length: 5.30 (5.10-5.50) mm.

Female

Head.
Antenna yellow-orange. Third antennal segment twice as long as second segment.
Arista with short hairs over entire length. Frons with short scattered hairs
which are largely the same colour as frons, at most slightly darker; convex, in
lateral view not distinctly projecting forwards at antennal implant. Chaetotaxy
normal for subgenus.

Thorax.
Postpronotum pale yellow, unspotted. Ground colour of mesonotum pale with
yellow-orange tinge. Chaetotaxy normal for subgenus. Scapular setae pale. Two
anepisternal bristles. Scutellum white basally, otherwise yellow with three
black separate markings apically; basally without dark spots, only slight
darker colouration. Subscutellum three black spots at same position as apical
scutellar spots.

Legs
yellow; setation typical for subgenus, mainly pale especially on femora.
Ventral, posterior and posterodorsal rows on fore femur pale. Ventral spines
dark reddish.

Wing
bands with extensively yellow markings. Banding as in fig. 1a, marginal band
continuous; cubital band free, medial band absent; crossvein r-m before middle
of discal cell (in paratype at anterior third). Crossvein dm-cu posteriorly
more inwards than anteriorly.

Abdomen.
Yellow. Setation and banding typical for subgenus. Oviscape about twice as long
as abdominal terga 3-6 combined.�

Male

Unknown.

(Description
after De Meyer, 1998)
